# Superchiral near fields detect virus structure

**DOI:** 10.1038/s41377-020-00433-1

**Published:** 2020-12-01

**Authors:** Tarun Kakkar, Chantal Keijzer, Marion Rodier, Tatyana Bukharova, Michael Taliansky, Andrew J. Love, Joel J. Milner, Affar S. Karimullah, Laurence D. Barron, Nikolaj Gadegaard, Adrian J. Lapthorn, Malcolm Kadodwala

**Affiliations:** 1 School of Chemistry, Joseph Black Building, University of Glasgow, Glasgow, G12 8QQ UK; 2 Institute of Molecular, Cell and Systems Biology and School of Life Sciences, University of Glasgow, G12 8QQ Glasgow, UK; 3 James Hutton Inst, Cell & Mol Sci, Dundee, DD2 5DA UK; 4 Shemyakin-Ovchinnikov Institute of Bioorganic Chemistry RAS, Moscow, 117997 Russia; 5 School of Engineering, Rankine Building, University of Glasgow, Glasgow, G12 8LT UK

**Keywords:** Circular dichroism, Photonic devices

## Abstract

Optical spectroscopy can be used to quickly characterise the structural properties of individual molecules. However, it cannot be applied to biological assemblies because light is generally blind to the spatial distribution of the component molecules. This insensitivity arises from the mismatch in length scales between the assemblies (a few tens of nm) and the wavelength of light required to excite chromophores (≥150 nm). Consequently, with conventional spectroscopy, ordered assemblies, such as the icosahedral capsids of viruses, appear to be indistinguishable isotropic spherical objects. This limits potential routes to rapid high-throughput portable detection appropriate for point-of-care diagnostics. Here, we demonstrate that chiral electromagnetic (EM) near fields, which have both enhanced chiral asymmetry (referred to as superchirality) and subwavelength spatial localisation (∼10 nm), can detect the icosahedral structure of virus capsids. Thus, they can detect both the presence and relative orientation of a bound virus capsid. To illustrate the potential uses of the exquisite structural sensitivity of subwavelength superchiral fields, we have used them to successfully detect virus particles in the complex milieu of blood serum.

## Bioimaging: Spotting viral signatures with ‘superchiral’ light

A technique that uses twisted light fields to detect biomolecular structures could find application as a low-cost clinical tool for screening viruses. The protein coatings around many viruses, such as the turnip yellow mosaic virus (TYMV), have complex polyhedral shapes that are difficult to resolve with conventional optical microscopes. Malcolm Kadodwala from the University of Glasgow and other colleagues in the United Kingdom now report that ‘superchiral’ light — localized fields generated by metal nanostructures that spiral as they travel — are sensitive to the asymmetric polyhedral of TYMV. By spectroscopic measurements of particle rotations in superchiral light at different frequencies, the team identified specific asymmetric signals that correlated to virus alignment on gold photonic substrates. This approach was then used to determine TYMV levels in human blood serum spiked with the virus.

## Introduction

One of the markers of the transition from chemistry to biology is when individual molecular building blocks self-assemble into complex biological architectures. Optical spectroscopy provides a means of characterising the static and dynamic structural properties of individual molecules through probing of quantised states. However, optical spectroscopy cannot generally do the same for molecular assemblies^1,2^. Thus, characterisation of the static and dynamic structural properties of biological assemblies is achieved through alternative techniques, diffraction and NMR, which lack the advantages of ease of use and rapidity of optical spectroscopy. In this work, we seek to span this length scale gap in the spectroscopic toolbox. We show that electromagnetic (EM) near fields of subwavelength extent that have enhanced chiral asymmetry (superchirality) can probe the structure of biomolecular assemblies. To validate this hypothesis, we used chiral near fields to sense the chiral structure of a model biological assembly, a plant virus with an icosahedral capsid (turnip yellow mosaic virus (TYMV)) and thus detect its relative alignment on a surface.

Spectroscopy is sensitive to the structure of individual free floating (i.e. orientationally averaged) molecules in solution because they have electronic and vibrational states that provide structurally sensitive spectroscopic fingerprints^3^. In general, when molecules aggregate into larger assemblies, the electronic states of the monomer are not perturbed, and therefore, the spectroscopic response reflects the monomer and not the aggregate structure^4^. There are exceptions to this, such as J-aggregates, which have a different spectroscopic response than the individual component monomer^4^. However, this requires wavefunction mixing, which occurs for relatively simple aromatic molecules, that creates new electronic states correlated with the structure of the aggregate that provide a spectroscopic fingerprint. This does not occur for structurally and compositionally more complex biological assemblies (e.g. virus capsids formed by the assembly of protein molecules). In the absence of electronic perturbations upon aggregation, spectroscopy can discriminate between molecular assemblies that are strongly anisotropic (e.g. rod-like structures with high aspect ratios) and other assemblies. This arises because anisotropic aggregates have molecular polarisabilities with respect to the molecular reference frame that are not equivalent. Consequently, polarisation-dependent spectroscopic techniques, such as oriented circular dichroism (CD)^5,6^, linear dichroism (LD)^7–9^ and polarised Raman^10^, can be useful. However, in the general case of aggregates that are not strongly anisotropic, alignment does not provide a route to additional information. For example, spectroscopy is insensitive to the details of the icosahedral structures adopted by a vast array of viruses. This is because the size of the capsid is much smaller than the wavelength of light required to excite the chromophores of the coat proteins (IR/Vis/UV). Consequently, the electric field is uniform throughout the virus particle, and the spectroscopic response is insensitive to the spatial distribution of the coat proteins (CPs) in the capsid. Effectively, to an IR/Vis/UV photon, icosahedral capsids are spherical objects. Hence, spectroscopic characterisation of virus capsids is limited to fingerprinting the secondary structure and folds of the coat protein subunits with techniques such as UV/VIS CD^11^, vibrational CD (VCD)^12^ and Raman optical activity (ROA)^13–16^.

Near fields are localised nonpropagating EM fields created by light scattering from nanostructures. They vary spatially on a length scale 1–2 orders of magnitude smaller than the wavelength of light from which they are generated. Light scattering from chiral nanostructures creates near fields that in local regions of space, possess chiral asymmetries greater than the incident light, a property sometimes referred to as superchirality^17–20^. These chiral near fields display strong spatial variations of both the intensity and chiral asymmetry on length scales ≤ the size of the virus capsid. It is this combination of subwavelength localisation and enhanced chiral asymmetry to which the enhanced structural sensitivity is ascribed.

To validate the structural sensitivity of chiral near fields, we demonstrate a dependency of the interaction of a superchiral near field on the alignment of the TYMV particle. Any dependency on alignment provides definitive evidence that the superchiral near fields are sensitive to the structural details of the icosahedral virus capsid. Our results presage a spectroscopic approach for characterising the static and dynamic structural properties of viruses.

**Fig. 1:**
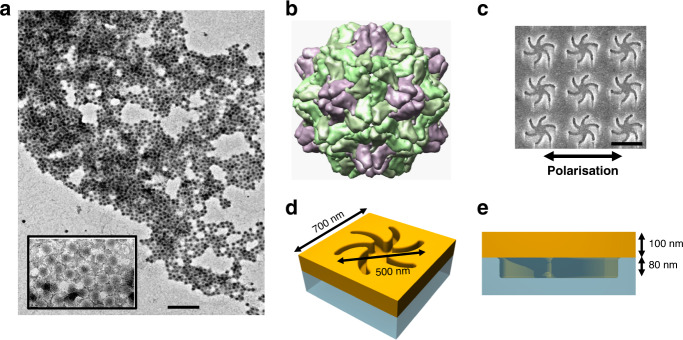
Graphics of the virus and nanostructure are shown. **a** SEM image of the TYMV particles, scale bar 200nm, with the inset showing an amplified view. **b** Representation of the TYMV particle, with the pentamers and hexamers highlighted in purple and green, respectively. Schematic of the **c** top and **d** side of the TPS metafilm. **e** SEM image of the TPS metafilm, scale bar 500nm. The polarisation direction used for ORD is shown.

Plant viruses are ideal for this study because they are readily available in large quantities, are non-pathogenic to humans, have well characterised structures, and can be immobilised onto a surface with relatively well-defined orientations using biochemical techniques. TYMV has an unenveloped icosahedral capsid with a quasi-symmetry of *T* = 3, assembled from 180 subunits of an identical sequence (with 60 subunits forming 12 pentamers and 120 subunits forming 20 hexamers) coat protein (CP) with a molecular mass of 20,600 kDa and a diameter of 28 nm (Fig. 1). RNA is organised within the interior of the protein capsid with little or no penetration into the coat protein^21^ and exhibits icosahedral order^22^. Virus particles display a chiral structure on two length scales: the secondary and tertiary structures of protein subunits and the quaternary structure of the icosahedral capsid. The chirality of the icosahedral capsid assembly (point group I) is derived from the mirror symmetry breaking of the protein subunits.

**Fig. 2:**
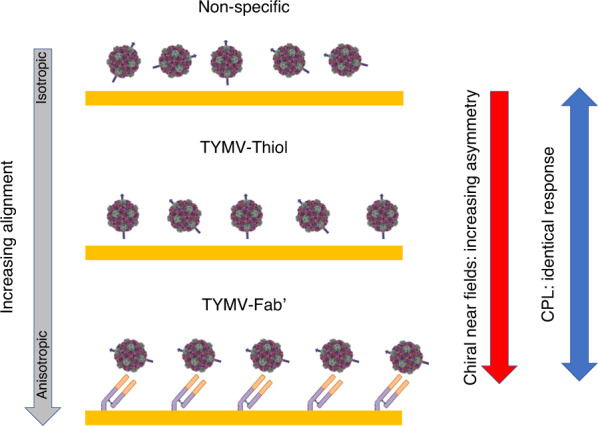
Illustration of the concept proposed in the current study. The arrows associated with the virus particles are representative of a specific well-defined axis. While chiral near fields can detect virus alignment, circularly polarised light (CPL) cannot.

Measurements were carried out using TYMV particles adsorbed from solution directly onto the substrate. It is assumed that TYMV will nonspecifically bind to the substrate, producing random orientations, hence creating an overall isotropic distribution. Varying levels of alignment of TYMV have been achieved using two surface immobilisation strategies (Fig. 2). The first approach involves functionalising lysines located on the pentamers and hexamers of the capsid surface (see supplementary information) with thiol groups that can bind the virus to a Au surface. This approach has a higher level of alignment than nonspecific binding with a mixture of capsids with either the pentamer or hexamer next to the surface (in a 3:5 ratio). These thiolated particles will subsequently be referred to as TYMV-Thiol. The second approach utilises surface immobilised fragment antibodies (Fab’) to specifically orient the virus particles with respect to the surface. This method produces the greatest level of alignment

Gold metafilms formed on a nanostructured polycarbonate template were used in this study^23^. They were ~100-nm thick and consisted of either left-handed (LH) or right-handed (RH) “shuriken” shaped indentations (Fig. 1) that possessed six-fold rotational symmetry and were arranged in a square lattice. These substrates are referred to as “template plasmonic substrates” (TPSs) for brevity. The nanoscale indentations in the surface polycarbonate substrate have a depth of ~80 nm, are 500 nm in diameter from arm to arm, and have a pitch of 700 nm. A detailed discussion of the chiral and optical properties of these substrates can be found elsewhere^24^.

### Theory

**Fig. 3:**
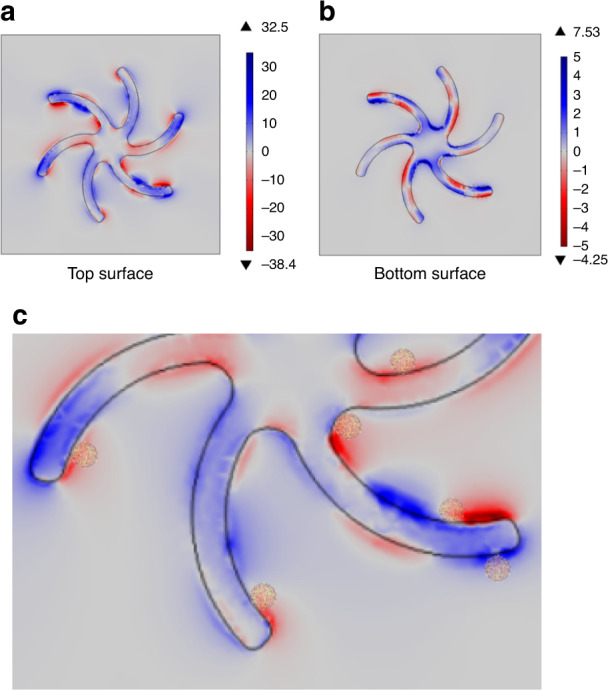
The chiral asymmetry of the near field can be quantified using a parameter known as optical chirality (C); values >1 indicate enhanced asymmetry (superchirality) compared to circularly polarised light. This figure shows numerical simulations of the C values for fields at the **a** top and **b** bottom surface of the shuriken structures. **c** Magnified region of the top surface illustrating the relative sizes of the fields, nanostructure and virus particles.

The chiral asymmetry of a near field of frequency ω can be parametrised by the optical chirality density parameter (*C*)^19,25^

1 $$C = \frac{1}{2}\left( {{\boldsymbol{D}} \cdot {\dot{\boldsymbol B}} - {\boldsymbol{B}} \cdot {\dot{\boldsymbol D}}} \right)$$

where **D** is the displacement field, **B** is the magnetic induction and **D** and **B** are their respective time derivatives. In free space, the optical chirality density is conserved; however, in localised regions of space, *C* can be higher than that of equivalent circularly polarised light (CPL), a property that has been referred to as superchirality^19,20^. Numerical simulations have been performed to calculate the *C* of the near fields, as shown in Fig. 3a, which locally display superchirality (C > 1). Both the spatial extent and chiral asymmetries vary on a length scale comparable to the size of TYMV (Fig. 3b).

The interaction of EM fields with chiral dielectrics (such as biomaterials) can be understood through the following constitutive equations:

2 $${\boldsymbol{D}} = \varepsilon _o\varepsilon _r{\boldsymbol{E}} + i\xi {\boldsymbol{B}}$$

3 $${\boldsymbol{H}} = {\boldsymbol{B}}/\mu _0\mu _r + i\xi {\boldsymbol{E}}$$

Here, (*ε*_*r*_) *ε*_*o*_ is the (relative) permittivity of free space, and (*μ*_*r*_) *μ*_0_ is the (relative) permeability of free space. ***E*** is the complex electric field, and ***H*** is the magnetic field. *ξ*(*λ*) is a wavelength-dependent second rank complex tensor describing chiral molecular properties, the sign of which is dependent on the handedness, and it is zero for achiral media. In the case that electric dipole–magnetic dipole interactions are the sole contributor to optical activity, *ξ*(*λ*) takes the form

4 $$\left| {\begin{array}{*{20}{c}} {\xi _{xx}\left( \lambda \right)} & 0 & 0 \\ 0 & {\xi _{yy}\left( \lambda \right)} & 0 \\ 0 & 0 & {\xi _{zz}\left( \lambda \right)} \end{array}} \right|$$

For the TYMV particle, the chiral response is derived from a combination of the assembled coat proteins and the RNA,

5 $$\xi _{{\mathrm{aa}}}^{{\mathrm{eff}}}\left( \lambda \right) = \xi _{{\mathrm{aa}}}\left( \lambda \right)^{{\mathrm{Capsid}}} + \xi _{{\mathrm{aa}}}\left( \lambda \right)^{{\mathrm{RNA}}}$$

where $\xi _{{\mathrm{aa}}}^{{\mathrm{eff}}}\left( \lambda \right)$ (*a* = *x*, *y*, *z*) are the effective tensor elements of the virus particle, and $\xi _{{\mathrm{aa}}}\left( \lambda \right)^{{\mathrm{Capsid}}}$ and $\xi _{{\mathrm{aa}}}\left( \lambda \right)^{{\mathrm{RNA}}}$ are the individual contributions of capsid and RNA;

6 $$\xi _{{\mathrm{aa}}}^{\,}\left( \lambda \right)^{{\mathrm{Capsid}}} = \mathop {\sum }\limits_{i = 1}^n \left( {\xi _{{\mathrm{aa}}}\left( \lambda \right)^{{\mathrm{Protein}}}} \right)_i$$

$\xi _{{\mathrm{aa}}}\left( \lambda \right)^{{\mathrm{Protein}}}$ are the tensor elements for individual protein subunits, and *n* = 180. In the case of TYMV, both $\xi _{{\mathrm{aa}}}\left( \lambda \right)^{{\mathrm{Capsid}}}$ and $\xi _{{\mathrm{aa}}}\left( \lambda \right)^{{\mathrm{RNA}}}$ have identical symmetry properties, both reflecting that of the *T* *=* *3* icosahedron.

For the case of interaction with light,

7 $$\xi _{zz}^{{\mathrm{eff}}}\left( \lambda \right) = \xi _{yy}^{{\mathrm{eff}}}\left( \lambda \right) = \xi _{xx}^{{\mathrm{eff}}}\left( \lambda \right)$$

for the icosahedral TYMV capsid. However, for the case of near fields with subwavelength spatial extent, for capsids aligned on a surface,

8 $$\xi _{zz}^{{\mathrm{eff}}}\left( \lambda \right) \gg \xi _{yy}^{{\mathrm{eff}}}\left( \lambda \right) = \xi _{xx}^{{\mathrm{eff}}}\left( \lambda \right)$$

Previously, the effects of the influence of chiral dielectrics, such as biomolecular layers, on the optical properties of chiral plasmonic materials were modelled using numerical EM simulations^24,26–29^. Constitutive Eqs. (2) and (3) were used in these simulations, and it was assumed that the chiral dielectric layers were continuous unstructured slabs. To account for the anisotropic/isotropic material properties, the relationships in Eqs. (7) and (8) were used. These equations have been used to simulate anisotropic^27,30^ and isotropic^24,26,28,30^ layers on chiral structures. These previous studies demonstrated that anisotropic layers induce larger asymmetries in the optical properties of LH and RH plasmonic structures than isotropic layers. Due to computational limitations, we cannot numerically simulate the interaction of chiral near fields with the nanoscale icosahedral virus capsids. However, the above theory provides a framework for understanding the presented experimental results in terms of the level of structural anisotropy within the virus layer. The concept of isotropic and anisotropic layers in the context of immobilised viruses is illustrated in Fig. 2. An isotropic layer arises when an ensemble of TYMVs adopt random orientations on the surface. An anisotropic layer is one in which TYMV has a well-defined alignment with respect to the surface.

In the current study, we have focussed on the asymmetry induced in the ORD spectra, an approach used in previous experimental^17,31^ and modelling^28^ studies, which has been parametrised using

9 $${\Delta} {\Delta} \lambda = {\mathrm{{\Delta} }}\lambda _{RH} - {\mathrm{{\Delta} }}\lambda _{LH}$$

where Δλ_*LH/RH*_ are the shifts induced (compared to a reference) in the position of the bisignate ORD peaks for left-handed (LH) and right-handed (RH) structures by the introduction of a chiral dielectric (TYMV). If there is a nonchiral change in the dielectric environment of the near field region, then ΔΔλ = 0. In this study, we have derived ΔΔλ from the two extremes of the bisignate line shape referred to as peaks 1 and 2, which are labelled ΔΔλ_1_ and ΔΔλ_2,_ respectively.

## Results

**Fig. 4:**
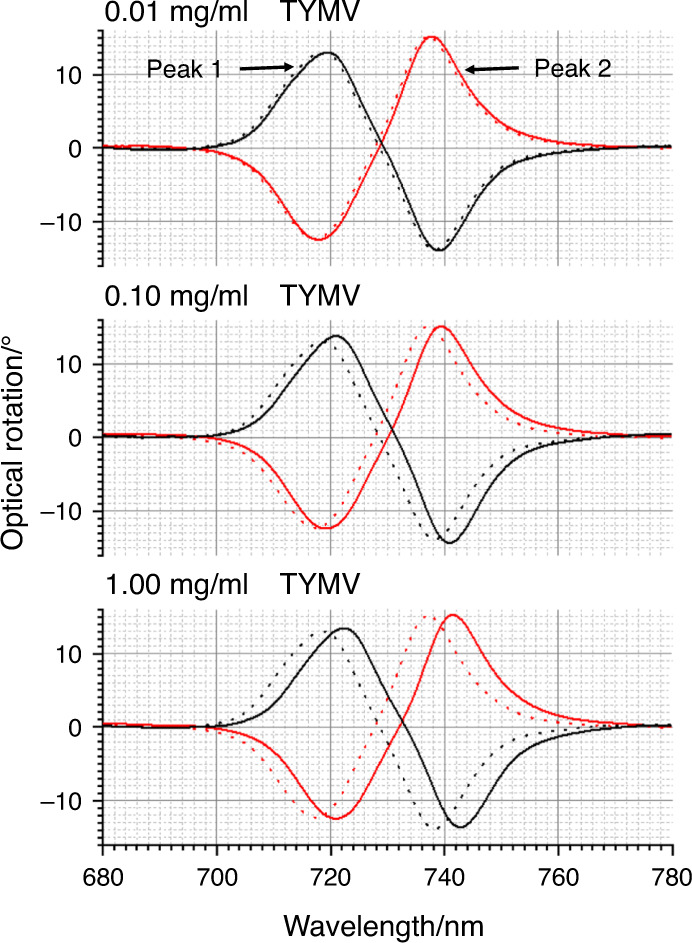
ORD spectra collected from LH (black) and RH (red) TPSs; this convention is used in all subsequent figures. The nested spectra collected for unfunctionalised TPSs in buffer (dashed) and those exposed to 0.01, 0.10 and 1.00 mg/ml TYMV in buffered solutions (solid). Peaks 1 and 2 from which the asymmetry parameters ΔΔλ_1_ and ΔΔλ_2_ are derived are labelled.

**Fig. 5:**
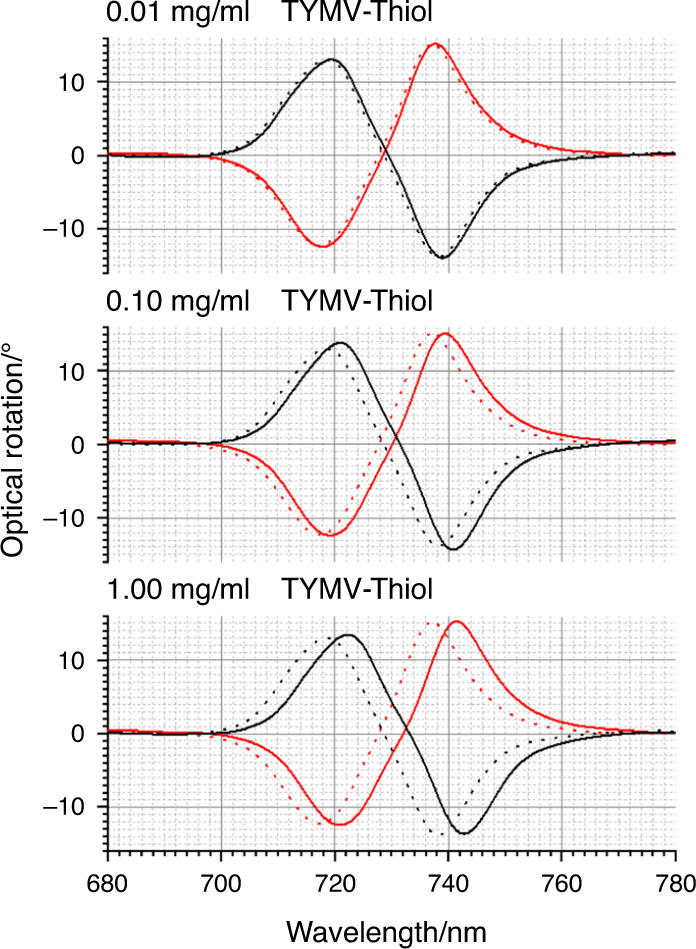
ORD data for TYMV-Thiol are shown. Nested spectra collected for unfunctionalised TPSs in buffer (dashed) and those exposed to 0.01, 0.10 and 1.00mg/ml TYMV-Thiol in buffered solutions (solid).

**Fig. 6:**
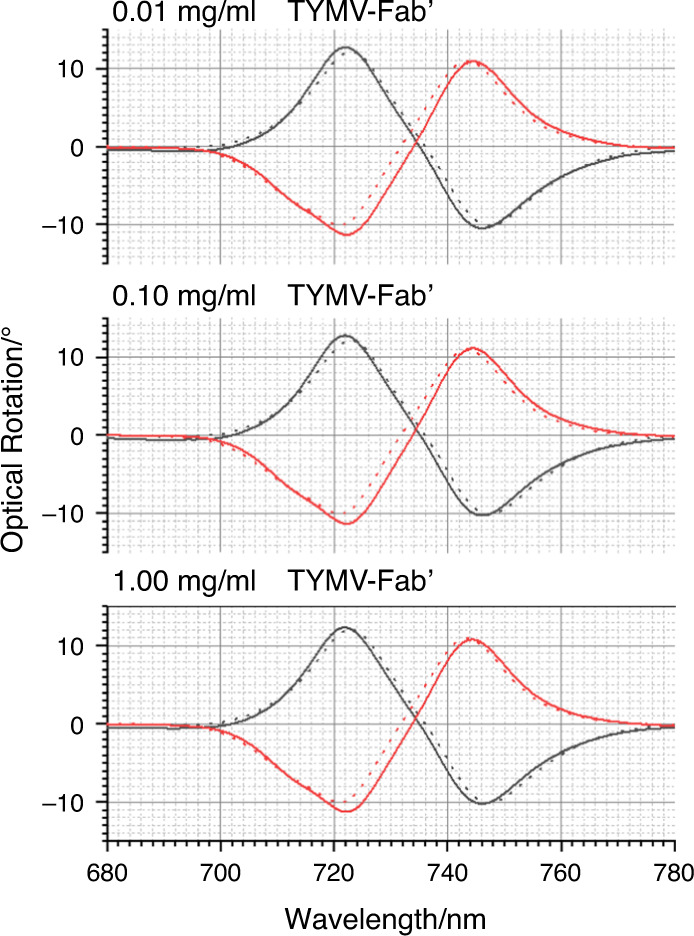
ORD data for TYMV bond to the mixed-Fab’ layers are shown. Nested spectra collected for mixed-Fab’ layer-functionalised TPSs in buffer (dashed) compared with those exposed to 0.01, 0.10 and 1.00mg/ml TYMV in buffered solutions (solid).

**Fig. 7:**
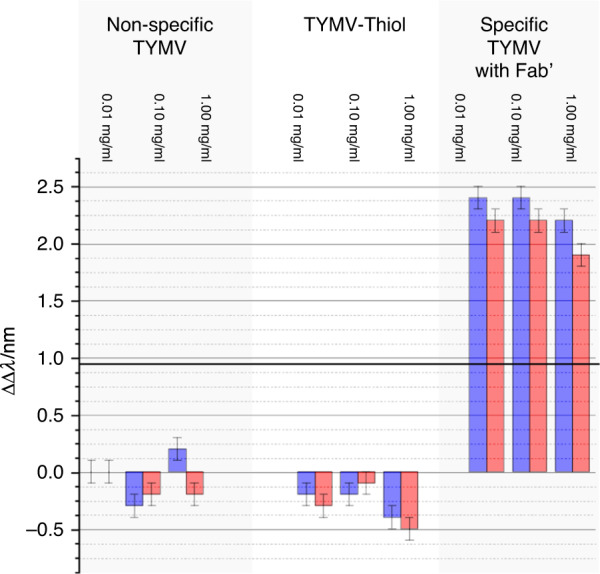
Asymmetry parameters ΔΔλ1 (red) and ΔΔλ2 (blue) derived from the data displayed in Figs. 4–6. Error bars indicate the standard error of the mean (*n* = 5).

The optical properties of the TPS are sensitive to chiral materials, displaying equal and opposite asymmetries in optical properties when exposed to molecular enantiomers^24^. Figure 4 shows the optical rotatory dispersion (ORD) spectra collected from LH and RH TPSs immersed in PBS buffer. The ORD spectra display a bisignate line shape, which, as expected, switches sign between the LH and RH structures. ORD spectra for TYMV nonspecifically bound to unfunctionalised TPSs, TYMV-Thiol and TYMV specifically bound to the mixed-Fab’ layer are shown in Figs. 4–6. The corresponding ΔΔλ_1,2_ parameters derived from these data are displayed in Fig. 7. The asymmetry parameters for nonspecifically bound TYMV and TYMV-Thiol are calculated relative to the positions of the ORD resonances for unfunctionalised TPSs in buffer, while the specifically bound TYMV shifts are relative to the functionalised layer. Data were obtained for three types of virus layers deposited from solutions that contained 0.01, 0.10 and 1.00 mg/ml TYMV.

Turning first to the nonspecific case of TYMV binding directly to Au (Fig. 4), greater amounts of virus adsorb with increasing concentration, based on the average shift of peak 1 $({\Delta} \lambda = {\mathrm{{\Delta} }}\lambda _{LH} + {\mathrm{{\Delta} }}\lambda _{RH}/2)$ (Supplementary Fig. 3). However, the magnitudes of the asymmetries are small, being just greater than the standard error. The small asymmetries are consistent with the experimental and modelling results of previous studies on nonspecifically bound, structurally isotropic proteins on the same TPSs^27,30^.

The binding of TYMV-Thiol (Fig. 5) is similar to that of the unthiolated case, with similar amounts deposited at the three concentrations (Supplementary Fig. 4). The level of asymmetry is higher than that for TYMV, with a consistent (negative) asymmetry being observed at the three concentrations studied. This is indicative of the chiral field detecting a degree of structural anisotropy in the TYMV-Thiol layer caused by a level of preferential alignment.

The mixed-Fab’ layers in isolation produce asymmetries in the ORD spectra, which is indicative of the expected well-defined orientation of the immobilised Fab’ (Supplementary Fig. 5). The level of asymmetry is comparable to that obtained in a previous study of proteins bound in specific orientations, albeit using a His-Tag immobilisation strategy^31^.

Significantly greater asymmetries are observed for specifically bound TYMV than for both nonspecifically bound TYMV and TYMV-Thiol (Fig. 6). The amount of TYMV adsorbed onto the surface cannot be accurately gauged from the Δλ_AV_ values due to the large asymmetries produced. Indeed, the values of Δλ_RH_ and Δλ_LH_ for peaks 1 and 2 have similar magnitudes but opposite signs. Similar ΔΔλ_1,2_ values are observed for the three concentrations used, indicating that the amounts of specifically bound TYMV are similar for all three concentrations. This is to be expected, as the amount of immobilised TYMV is controlled by the strength of the antigen-antibody (TYMV-Fab’) interaction, defined by the equilibrium dissociation constant (K_d_). The typical K_d_ values for antigen-antibody binding fall in the range of 10–100 pM^32^. Because the concentrations of TYMV used here, 10–100 nM, are much greater than K_d,_ saturated layers are achieved on the surface in this study. The different signs of ΔΔλ_1,2_ for TYMV-Thiol and TYMV-Fab’ can be attributed to the relative contributions of the RNA and protein terms in Eq. (5).

**Fig. 8:**
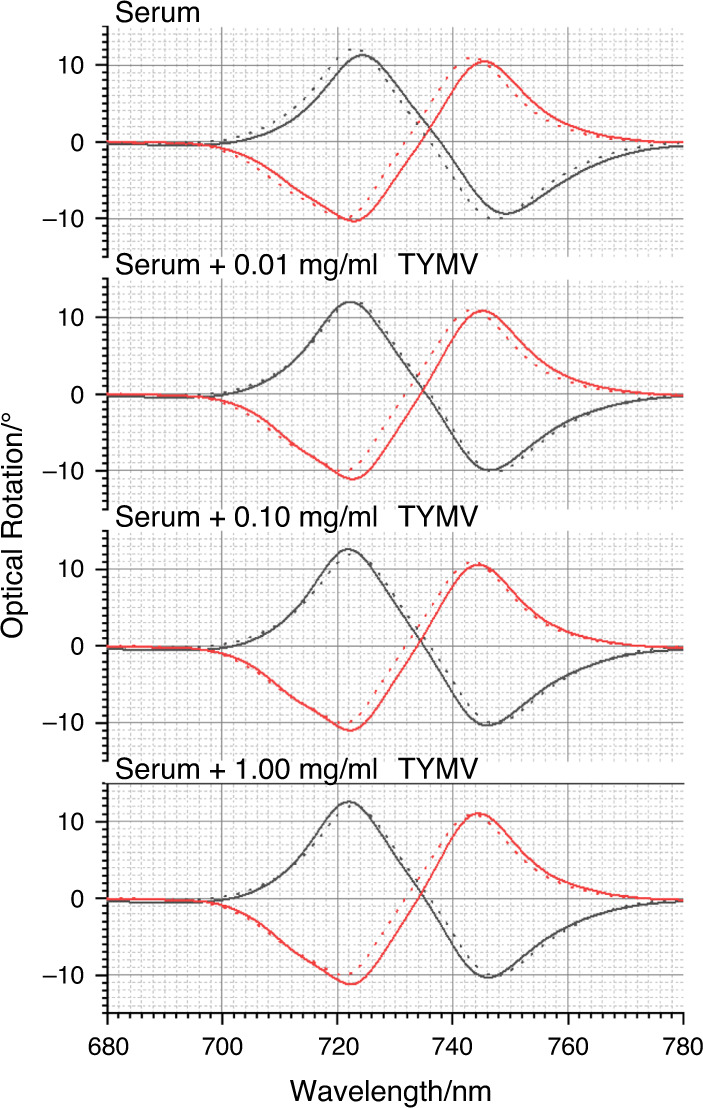
ORD data for TYMV bond to the mixed-Fab’ layers in the presence of serum are shown. Nested spectra collected for mixed-Fab’ layer-functionalised TPSs in buffer (dashed) compared with those exposed to serum, 0.01, 0.10 and 1.00mg/ml TYMV in serum (solid).

**Fig. 9:**
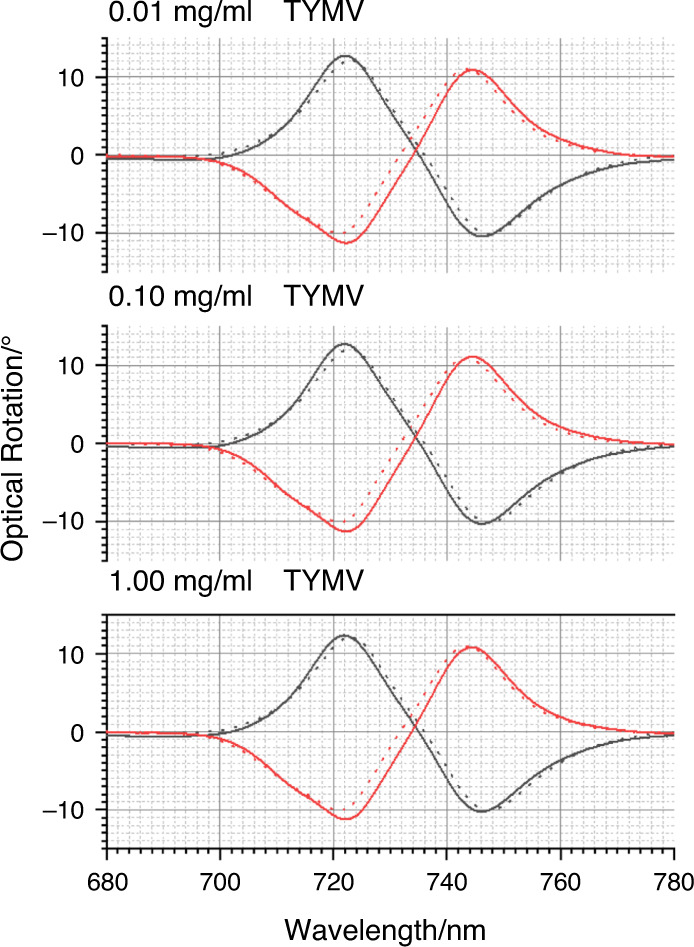
Spectra (solid) collected in serum for functionalised TPSs exposed to serum spiked with TYMV washed with copious serum. Functionalised TPSs in buffer (dashed) are shown for comparison.

**Fig. 10:**
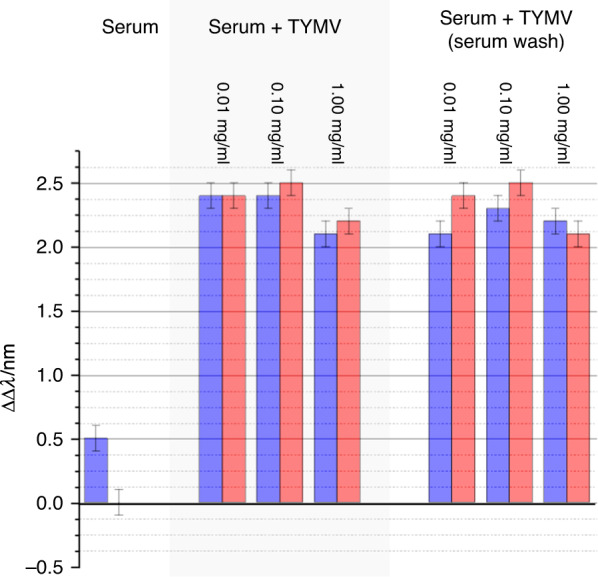
Asymmetry parameters ΔΔλ1 (red) and ΔΔλ2 (blue) derived from the data displayed in Figs. 8 and 9. Error bars indicate the standard error of the mean (*n* = 5).

To illustrate a potential application of the enhanced structural sensitivity of superchiral near fields, we used them to detect TYMV spiked into blood serum. Serum is a complex biological fluid comprising all the components of blood apart from blood cells and clotting agents. This complex milieu contains >1000 components, spanning nine orders of magnitude in concentration. It includes many different types of chiral molecules, such as serum proteins, antibodies, antigens, hormones and sugars. When a TPS with mixed-Fab’ layers is exposed to serum, some serum proteins will nonspecifically interact with the Fab’ component^30^. This arises because the specificity of Fab’ is to some extent degraded by immobilisation on the Au surface of the TPS. Consequently, when immersed in serum, the functionalised TPS surface will be covered in a disordered, probably multicomponent protein layer, often referred to as a protein corona. The thickness of this layer can be estimated to be equal to the molecular dimensions of a constituent protein (∼10 nm). When a functionalised TPS is immersed in serum spiked with TYMV, the virus particle will displace the nonspecifically bound layer. TPSs functionalised with the mixed-Fab’ layers were exposed to serum spiked with TYMV (0.01, 0.10 and 1.00 mg/ml) plus a control of nonspiked serum for 120 min, after which ORD spectra were collected. Subsequently, to remove any potentially nonspecifically bound TYMV, the TPSs were washed in copious amounts of nonspiked serum, and then, ORD spectra were collected in the presence of nonspiked serum. The ORD spectra for these two experiments are shown in Figs. 8 and 9. The ΔΔλ_1,2_ parameters, shown in Fig. 10, were calculated relative to the mixed-Fab’ layer in buffer. Nonspiked serum gave rise to a small asymmetry, which can be attributed to the structurally disordered (isotropic) blood protein—Fab’ complexes formed through nonspecific interactions. For TYMV-spiked serum, significant asymmetries were observed, which are all within the experimental error of the values obtained for the virus immobilised in buffer. There was no significant difference between the data collected in the presence of spiked serum and after replacement with unspiked serum. These measurements indicate that structurally well-defined TYMV-Fab’ complexes are formed even in a serum milieu.

## Discussion

The primary and most significant result of this work is the sensitivity of chiral near fields to the higher order (quaternary) structure of the virus capsid, established by the correlation between the level of orientational order and the magnitude of the optical asymmetries. The inherent novelty of the current work lies in the ability of chiral near fields to detect the icosahedral shape of a virus, a complex self-assembled biological aggregate, which is invisible to conventional spectroscopic phenomena that utilise CPL. This goes beyond the previous examples of using chiral near fields to probe/detect the lower-order structure of simpler biomolecules. Clearly, the reported phenomenon does not provide the rich structural information of high-resolution crystallography. However, the icosahedral TYMV capsid is sensed by the chiral fields, rather than appearing as an isotropic spherical object as in optical spectroscopic techniques. In effect, the chiral near field can detect not only the presence of a virus particle but also its orientation. This unique capability can be used to enhance the effectiveness of immunoassays for pathogenic virus detection. A virus particle binds to an immobilised antibody element via an epitope on the capsid surface. Consequently, the relative orientation of the virus is determined by where the epitope is on the capsid surface. The novel structural sensitivity of chiral near fields provides an additional discrimination mechanism that could offer a means of differentiating between, for instance, two closely related virus pathogens that may either bind to a recognition element via different epitopes or produce specific and nonspecific binding. The combination of the novel structural sensitivity with the binary binding/nonbinding functionality of conventional immunoassays offers the potential to mitigate false positive results.

If one wished, the reported experiments could be performed using a combination of expensive quartz substrates fabricated using electron beam lithography and commercially available spectrometers^17^. However, given the typical spectral acquisition times of ∼10 min, combining our low-cost disposable polycarbonate substrates with portable reflectance polarimetry^33^ would create an effective field diagnostic technology.

In summary, using TYMV as a model, it has been demonstrated that superchiral EM fields with subwavelength spatial extent are, in contrast to normal light, sensitive to the higher order icosahedral symmetry of the virus capsid. We believe that in the context of the biophysical toolbox, the sensitivity of superchiral near fields is best exploited as a spectroscopic “triaging” tool, providing speedy low-resolution assessment of materials, which, if required, can subsequently be studied in more detail with costly low-throughput and high-resolution techniques. The ability of superchiral near fields to detect virus particles within serum provides a proof-of concept of the potential applications, presaging a novel label-free simple spectroscopic measurement for immunoassays for detecting viruses. The enhanced structural incisiveness of superchiral fields provides an additional parameter for discriminating between target and off-target interactions with an immobilised recognition element.

## Materials and methods

### Optical rotatory dispersion (ORD) and reflectivity measurements

ORD spectra were collected using a custom-made polarimeter that measures the reflected light from our samples. The design is similar to a basic reflected light microscope with a tungsten halogen light source (Thorlabs), Glan-Thompson polarisers (Thorlabs) and a ×10 objective (Olympus). A camera (Thorlabs) is used to position the sample, and spectra are collected using a compact spectrometer (Ocean optics USB4000). ORD spectra are obtained using the Stokes method, and the intensity of light is measured at four analyser angles (0°, ±45° and 90°). LH and RH pairs of ORD spectra are collected in 10 min.

### Simulations

Electromagnetic (EM) simulations were performed using a commercial finite-element package (COMSOL v4.4, Wave Optics module). Periodic boundary conditions were used to emulate the metafilm arrays. Perfectly matched layer conditions were used above and below the input and output ports. Linearly polarised EM waves were applied at normal incidence onto the films. COMSOL uses the finite-element method to solve Maxwell’s equations for a specified geometry with the fields and optical chirality being measured at predefined surfaces above, within, and below the films.

### TYMV purification

*Brassica rapa* var*. pekinensis* plants were grown under greenhouse conditions at 21 °C. Turnip yellow mosaic virus (TYMV) in crude sap extract mixed with abrasive celite was rub inoculated onto a true leaf of each *Brassica rapa pekinensis* plant grown to the first two true leaf stage of development. After 4 weeks, the plants developed strong systemic symptoms and were harvested, and total TYMV was isolated according to the protocols established by Leberman^34^ with the modifications suggested by Katouzian–Safadi and Berthet–Colominas^35^, with high speed centrifugations being carried out in a Beckman SW 41Ti rotor at 28,000 rpm for 3 h. The pelleted virus was resuspended in 10 mM Tris HCI pH 7.5 with 0.1 M EDTA and was subjected to CsCl density centrifugation in a gradient to obtain samples with densities of 1.26, 1.36 and 1.46, which were loaded into a 13.2 ml Thinwall Ultra-Clear tube (Beckman Coulter) and centrifuged in a SW 41Ti rotor at 28,000 rpm for 3 h. The upper band corresponding to the natural top component (empty virus) and the lower band corresponding to the bottom component (virus containing genome) were visualised under white light and isolated using a needle attached to a syringe. The samples were diluted and centrifuged in an SW 41Ti rotor as before to a pellet and were resuspended in an appropriate buffer, centrifuged to a pellet and resuspended in the buffer again to remove CsCl. The concentrations of the TYMV forms were estimated according to the protocols of Tamburro et al.^36^.

### TYMV-thiol production

The thiol groups were attached to the lysines at the virus surface by means of N-hydroxysuccinimide (NHS) ester chemistry, also called amine-reactive cross linker chemistry. At physiological pH, the amines of a protein are positively charged and therefore sit at the protein surface. These amines then become available for conjugation reagents. A fluorometric thiol assay was used to confirm virus thiolation.

### TYMV-specific F(ab’)_2_ production

TYMV-specific rabbit polyclonal IgG was purchased from DSMZ (AS-0125), and antibody specificity against the TYMV coat protein (CP) was confirmed by western blot analysis (see Supplementary Fig. 2). TYMV-specific F(ab’)_2_ fragments were generated using the Pierce™ F(ab’)_2_ preparation kit (Thermo Fisher Scientific: 44988) following the manufacturer’s instructions. Briefly, a TYMV-specific IgG sample was loaded onto a prewashed Zeba Spin Column using digestion buffer (20 mM sodium acetate, pH 4.4; 0.05% sodium azide) and centrifuged at 5000 × *g* for 1 min (Eppendorf Centrifuge 5415D) to collect the desalted sample. The immobilised pepsin resin was prepared by adding 65 µL of the 50% slurry placed into the 0.8 µL spin column, followed by centrifugation at 5000 × *g* for 1 min. The buffer was discarded, and the resin was washed with 130 µL of digestion buffer, followed by centrifugation at 5000 × *g* for 1 min. The flow through was discarded, and 125 µL of desalted TYMV-IgG was loaded onto the spin column with a capped bottom containing the pepsin resin. The sample was briefly mixed using a vortex. The digestion reaction was incubated for 2 h using an end-over-end mixer in a static 37 °C incubator. Next, the bottom cap was removed, and the spin column was placed into a microcentrifuge tube. The sample was centrifuged at 5000 × *g* for 1 min to separate the digest from the immobilised pepsin. The resin was washed twice with 130 µL of PBS (0.1 M sodium phosphate, 0.15 M sodium chloride; pH 7.2) by centrifugation at 5000 × *g* for 1 min. The flow through containing F(ab’)_2_ fragments was collected, and the immobilised pepsin was discarded. To remove any undigested IgG, the F(ab’)_2_ fragments were further purified using a Nab protein A Plus Spin Column. The equilibrated column was washed twice with 400 µL of PBS by centrifugation at 5000 × *g* for 1 min. The bottom of the column was capped, and the sample was added to the column. The samples and resin were resuspended by inversion followed by a 10 min incubation at room temperature with an end-over-end mixer. Next, the column was placed in a new collection tube, and the flow through containing F(ab’)_2_ was collected by centrifugation at 5000 × *g* for 1 min. The column was washed twice with 200 µL of PBS by centrifugation at 5000 × *g* for 1 min, and both wash fractions were added to the sample. The protein concentration was determined using the Pierce™ BCA Protein assay kit (Thermo Fisher Scientific: 23225) following the manufacturer’s instructions. To assess digestion and purification, the samples were analysed by SDS-PAGE using non-reducing loading dye and NuPAGE™ 4–12% Bis-Tris protein gels (Thermo Fisher Scientific: NP0336BOX) and SimplyBlue™ SafeStain Invitrogen™.

### Antibody fragments

Polyclonal antibodies are an ensemble of antibodies that can recognise multiple epitopes on an antigen. A monoclonal antibody, by contrast, displays greater specificity, binding uniquely to a single epitope of a macromolecular antigen such as a protein. In the current study, we immobilised onto the TPS a fragment derived from polyclonal rabbit IgG that had been produced against TYMV, referred to as poly anti-TYMV-IgG. Western blot analysis was used to confirm antibody specificity for the 20 kDa TYMV-CP (Supplementary Fig. 1). Surface immobilised IgG has been used as a recognition element in previous studies of plasmonic-based sensor platforms. Immobilisation significantly degraded the performance of IgG. This is attributed to a combination of structural heterogeneity and the large size of IgG facilitating denaturisation upon adsorption. Loss of functionality was minimised by immobilising functionally active fragments of IgG rather than the whole molecule^37,38^. Immobilised Fab’ fragments adopt more homogenous adsorption structures and are less susceptible to denaturing compared to the whole IgG molecule^39,40^. The IgG was treated with pepsin to produce smaller 88 kDa F(ab’)_2_ fragments, with a small portion of the digest representing Fab’ fragments, most likely due to over-digestion (Supplementary Fig. 2). In addition, we suggest that the majority of the F(ab’)_2_ fragments will cleave into Fab’ fragments after immobilisation onto the TPS. The Fab’ fragments have free sulfhydryl moieties that facilitate attachment to the gold surfaces of the TPSs. Importantly, this produces a consistent attachment point for the poly anti-TYMV-Fab fragments and should significantly limit effects due to the binding orientation. To minimise the potential denaturing of the Fab’ fragment through interaction with the Au surface, it is coadsorbed with a thiol: triethylene glycol mono-11-mecaptoundecyl (EG-thiol)^41^. EG-thiol is a neutral spacer molecule with biorepellent properties, so any interactions between Fab’ molecules and a surface will be minimised^20,42^. This layer will subsequently be referred to as a mixed-Fab’ layer. When tested against buffered solutions of single proteins, similar mixed-Fab’ layers were observed to not only retain specificity to the original target but also display nonspecific interactions with other types of protein molecules^30^. The Fab’ will bind to a limited range of epitopes (or a single epitope) of TYMV, thereby generating a narrow distribution of orientations relative to the nonspecific interactions of the unfunctionalised surface.

### Virus adsorption from solution

The TPSs were enclosed in a 100 μL cell, with TYMV and TYMV-Thiol adsorbed from a 10 mM PBS buffer, pH = 7.4. Stock serum solutions with a total protein concentration of 60 mg ml^−1^ were produced by dissolving lyophilised human blood serum (ERM^©^ certified reference material, Sigma–Aldrich) in distilled water. Serum (both nonspiked and spiked) solutions with a concentration ranging from 1 mg ml^−1^ were produced by diluting the stock serum solutions with 10 mM PBS buffer, pH = 7.4. Spiked serum samples were produced by adding the relevant amount of TYMV to the 1 mg ml^−1^ serum solution.

## Supplementary information

Supplementary Information
