# Supplementary material for: Superchiral near fields detect virus structure
Source: Light Sci Appl. 2020 Dec 1;9:195. doi: 10.1038/s41377-020-00433-1 (PMC7705013; doi:10.1038/s41377-020-00433-1)
Supplement: Supplementary file 1 — Supplementary Information [file 41377_2020_433_MOESM1_ESM.docx]

**Supplementary Information**

Superchiral near fields detect virus structure.

Tarun Kakkar^1*^, Chantal Keijzer^1,2*^, Marion Rodier^1^, Tatyana Bukharova^3^, Michael Taliansky^3,4^, Andrew J. Love^3^, Joel J. Milner^2^, Affar S. Karimullah^1^, Laurence D. Barron^1^, Nikolaj Gadegaard^5^, Adrian J. Lapthorn^1^ and Malcolm Kadodwala^1*^

^1^School of Chemistry, Joseph Black Building, University of Glasgow, Glasgow, G12 8QQ, UK

^2^Institute of Molecular, Cell and Systems Biology and School of Life Sciences, University of Glasgow, G12 8QQ, UK

^3^James Hutton Inst, Cell & Mol Sci, Dundee, DD2 5DA, UK

^4^Shemyakin-Ovchinnikov Institute of Bioorganic Chemistry RAS, Moscow, 117997, Russia

^5^School of Engineering, Rankine Building, University of Glasgow, Glasgow, G12 8LT, UK.

**Corresponding Authors ***

malcolm.kadodwala@glasgow.ac.uk, tarun.kakkar2@gmail.com, keijzerchantal@gmail.com

**
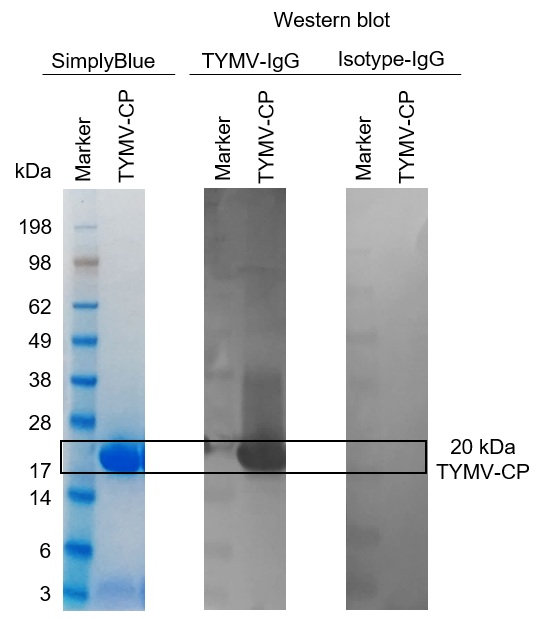
**

**Suppl.Fig.1:** **TYMV-specific rabbit polyclonal IgG antibody specificity against the TYMV coat protein confirmed by Western blot analysis.** Briefly, TYMV virus was diluted in NuPAGE™ LDS sample buffer (4x) (Thermo Fisher Scientific: NP0007) to a 1x solution and boiled for 15 min in a heat block at 95-100˚C. The samples were cooled down to RT and 1 µg of protein / lane was loaded onto a NuPAGE 4-12% Bis-Tris protein gel. The gel was run for ~45 min at 200 V and 440 mA at constant voltage in 1x NuPAGE™ MES SDS running buffer (Thermo Fisher Scientific: NP0002). The protein was blotted onto nitrocellulose membrane using the mini blot module (Invitrogen: B1000) at 10V for 1.5h at constant voltage. The membrane was transferred to a 50 mL Falcon tube and blocked with 7% dried skimmed milk (Marvel) dissolved in 1xTBS (Tris-buffered saline: 50 mM Tris-Cl, pH 7.5; 150 mM NaCl) for 30 min, at RT using a tube roller. The membrane was cut in half and the membranes were incubated with primary antibodies TYMV-IgG (DSMZ: AS-0125) or isotype-IgG (polyclonal rabbit IgG (Sigma-Aldrich: I5006-10MG). After incubation, the membranes were washed 3x5 min with 1xTBS+Tween-20 at 0.05% (TBS-T). Secondary antibody: anti-rabbit-IgG, (H+L), HRP conjugate (Promega: W4011) at 0.4 µg/mL final concentration in TBS-T was added to the membranes and incubated for 45 min at RT on a tube roller. After incubation, membranes were washed 5x5 min in TBS-T. Finally, 5 mL of 1-Step™ TMB-Blotting Substrate Solution (Thermo Scientific Pierce: 34018) at RT was added to each membrane and incubated at RT on a tube roller until bands develop. The reaction was stopped by removing the substrate and adding dH_2_O, followed by a final wash step with dH_2_O for another 5-10min.


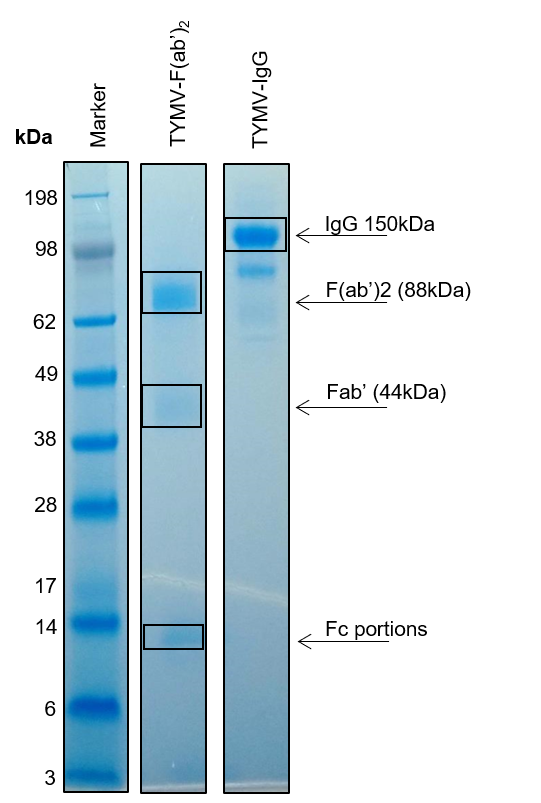


**Suppl.Fig.2: TYMV-specific F(ab’)_2_ fragment production._._**To assess digestion and purification, the samples were analysed by SDS-PAGE using non-reducing loading dye and NuPAGE™ 4-12% Bis-Tris protein gels. The gel was run for ~ 45 min at 200 V and 440 mA at constant voltage in 1x NuPAGE™ MES SDS running buffer. Next, the gel was washed with dH_2_O and stained using SimplyBlue™ SafeStain. The gel was microwaved until bands appear and incubated at RT on a plate shaker for another 15 min. Finally, the solution was removed and replaced with dH_2_O. Lane 1 shows the SeeBlue Plus2 pre-stained protein ladder, lane 2 shows the TYMV-specific F(ab’)_2_ fragments recovered after protein A column purification at 3 µg of protein/lane with a small fraction consisting of Fab’ and Fc portions. Lane 3 represents undigested TYMV-IgG antibody as a control for complete digestion and purification.

**
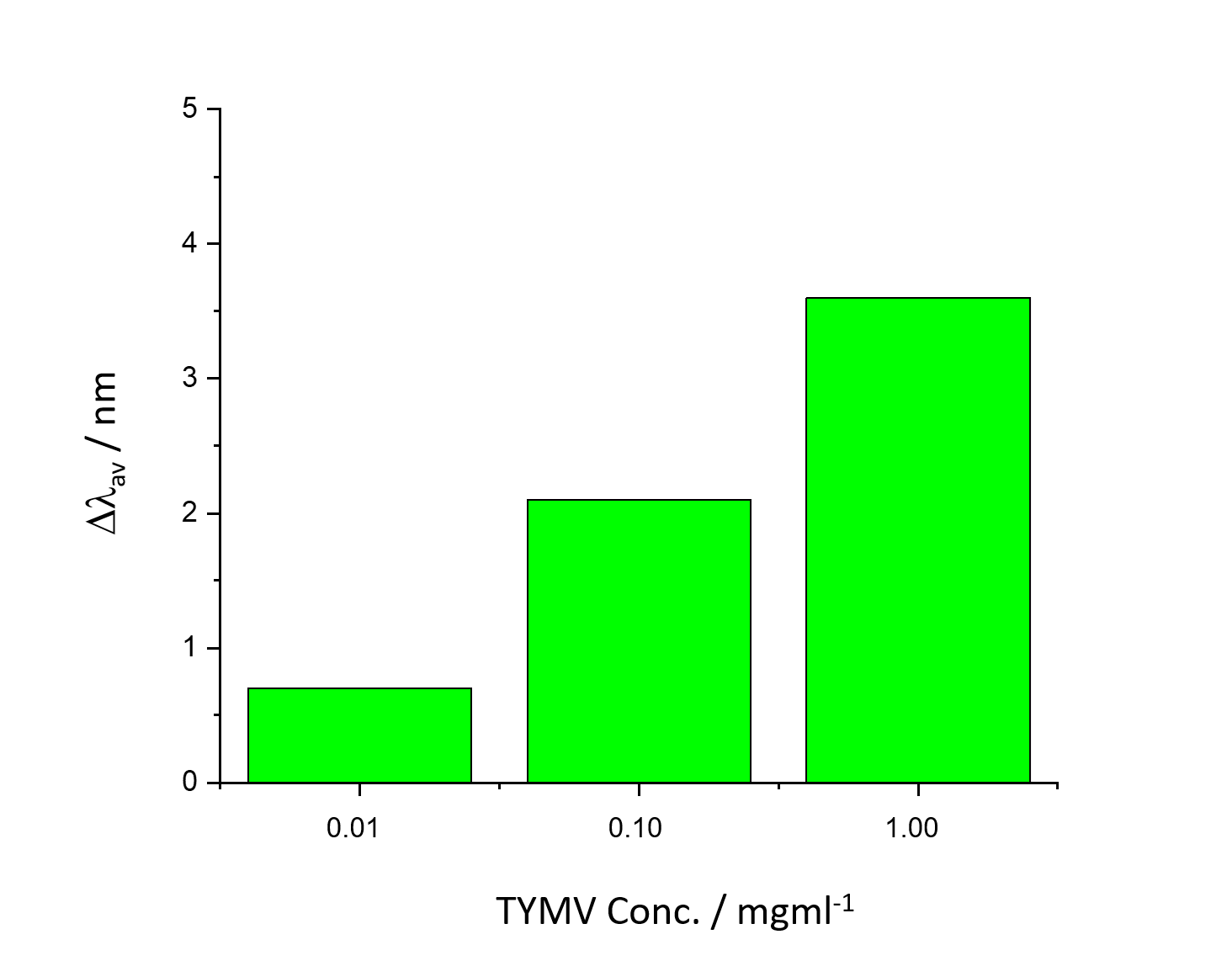
**

**Suppl.Fig.3:** **The amount of TYMV non-specifically bound to unfunctionalized TPSs.** The Δλ_Av_ values derived from the data in figure 4 is shown


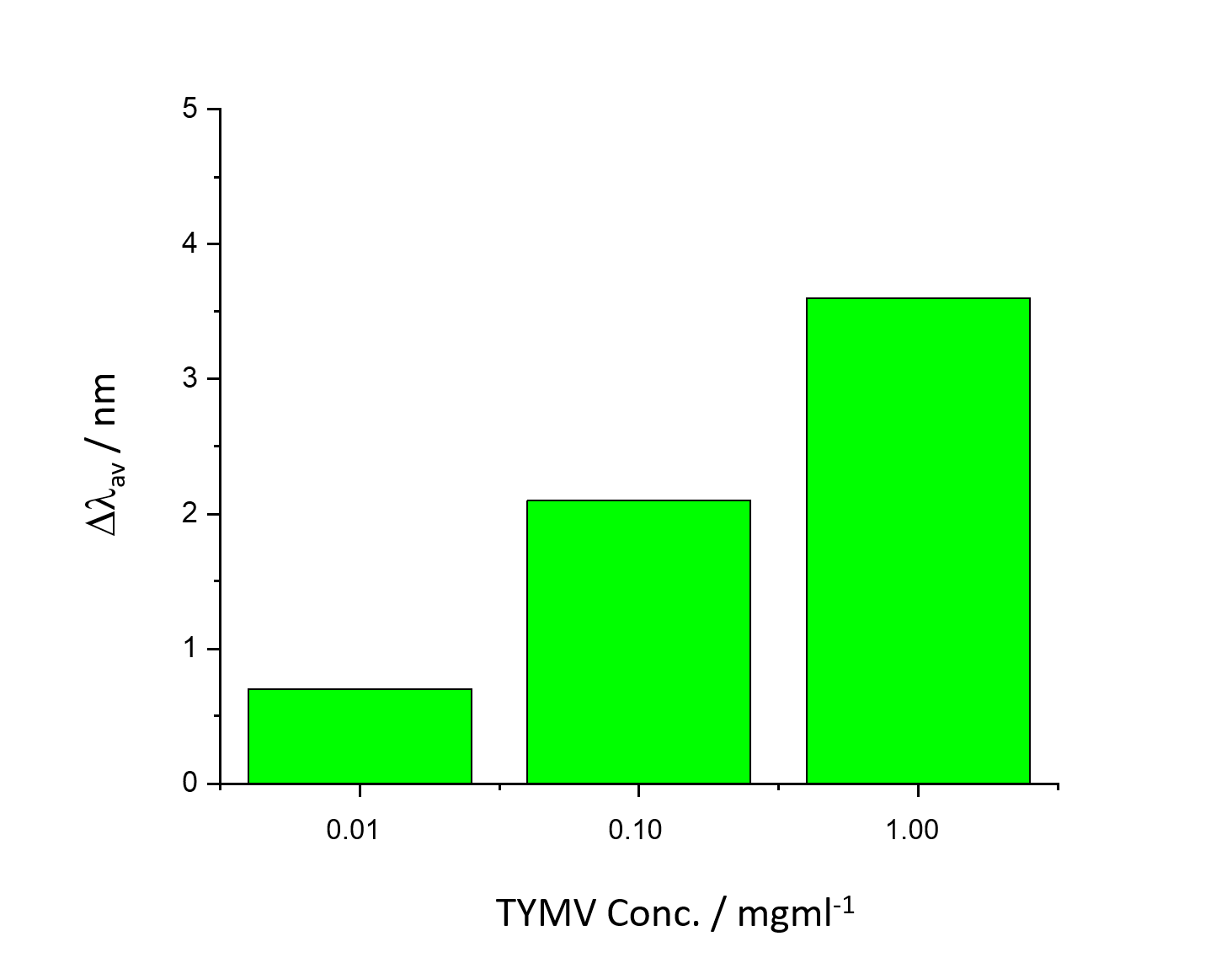


**Suppl.Fig.4:** **The amount of TYMV-Thiol bound to unfunctionalized TPSs.** The Δλ_Av_ values derived from the data in figure 5 is shown


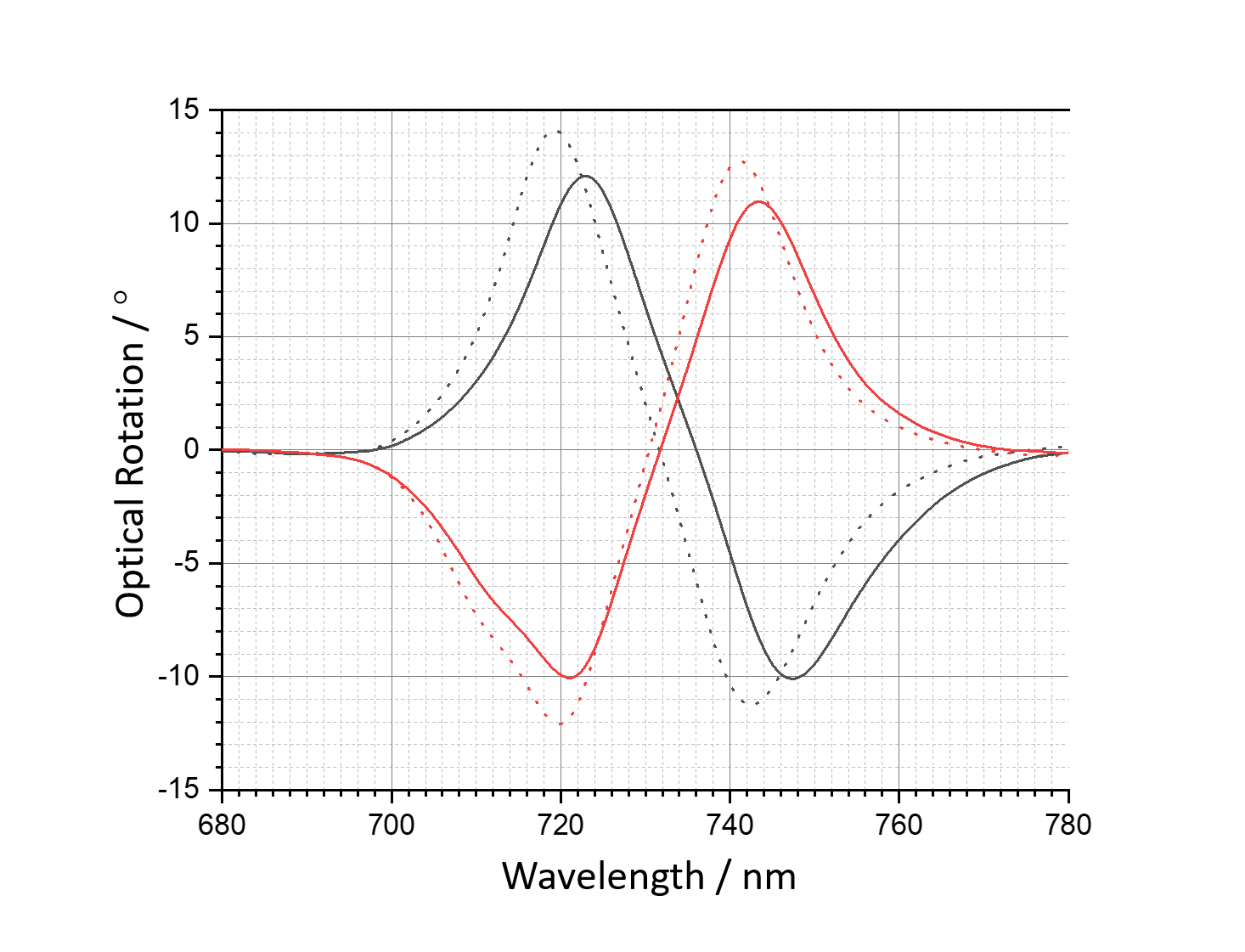


**Figure supp 5. Fab’ ORD Spectra.** Spectra collected from the TPSs functionalised with mixed-Fab’ layers (Solid) are compared to unfunctionalized TPSs (Dashed), both sets are immersed in buffer. The ΔΔλ_1_ and ΔΔλ_1_ values are -2.4±0.1 and -2.6±0.1 nm respectively.


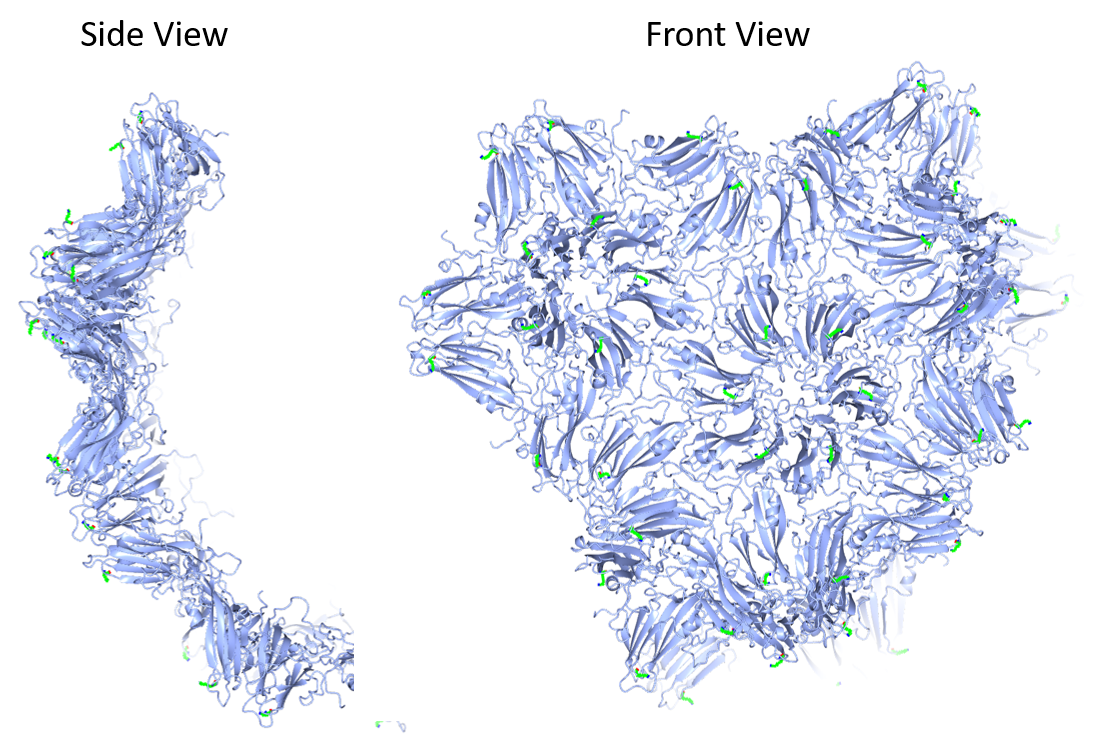


**Figure supp 6. Lysine positions.**  The side and front view of a portion of the TYMV capsid. The lysines functionalised by Thiols are highlighted in green. The thiols decorate the pentamers and hexamers of the capsid.
